# Tin and Gold

**Published:** 1908-11-15

**Authors:** C. E. Kells


### TIN AND GOLD.

BY C. E. KELLS, D.D.S.

Dr. Kells writing to the Editor of *Items of Interest* says that he notes on page 554 of the July issue the following statement: If tin and gold and hand pressure is so good why has it made so slight an impression upon the dental profession, considering that it has been preached and practiced for over a half of a century."

Now, why such a question! Is it not a well known fact that because a certain material or method of practice is not generally in vogue, it need not necessarily be condemned?

Tin and gold in combination (not necessarily "*and hand pressure*") have been used consistently by a sufficient number of successful operators who have seen it stand the test of time, to prove that it is no longer an experiment, and that it is not more generally used is to be regretted from the standpoint of the public.

Those who have used it and watched it for a quarter of a century or so, know full well that, when used where indicated, it has no equal for saving teeth. Those who have not used it successfully are in no position to judge of its value.

Inlays have been used sufficiently long to absolutely disprove the theory advanced by some, that decay would not recur about their margins.

The writer had the good fortune to be present at Niagara Falls in 1886 when Herr Herbst *introduced* the making of inlays from matrices to the dentists of this country, and has followed the practice in all its stages from that day to this.

But he does not believe that any system of inlays will ever entirely take the place of good *bona fide* old time gold, and gold and tin fillings.

He has gold fillings in his own teeth that were placed there about thirty-five years ago. And he frequently sees such fillings that have stood the stress of time for from forty to fifty years.

The trouble with the inlay is that it compels the sacrifice of a needless amount of tooth structure—that is, when it is a fad of the operator and used where fillings are indicated.

The easiest thing to do in the dental world is to make a Taggart inlay—I mean, of course, a poor one. Good inlays are usually very difficult propositions, and frequently more so than would be fillings in the same cases.

So please do not decry "tin and gold," even though you use Taggart inlays where the other material has long since demonstrated its value.

